# Revealing the mechanistic interactions of profenofos and captan pesticides with serum protein via biophysical and computational investigations

**DOI:** 10.1038/s41598-024-52169-2

**Published:** 2024-01-20

**Authors:** Kamonrat Phopin, Waralee Ruankham, Supaluk Prachayasittikul, Virapong Prachayasittikul, Tanawut Tantimongcolwat

**Affiliations:** 1 Center for Research Innovation and Biomedical Informatics, Faculty of Medical Technology, Mahidol University, Bangkok, 10700 Thailand; 2 Department of Clinical Microbiology and Applied Technology, Faculty of Medical Technology, Mahidol University, Bangkok, 10700 Thailand

**Keywords:** Biological fluorescence, Molecular biophysics, Drug development, Environmental sciences, Biochemistry, Chemical safety

## Abstract

Profenofos (PF) and captan (CT) are among the most utilized organophosphorus insecticides and phthalimide fungicides, respectively. To elucidate the physicochemical and influential toxicokinetic factors, the mechanistic interactions of serum albumin and either PF or CT were carried out in the current study using a series of spectroscopy and computational analyses. Both PF and CT could bind to bovine serum albumin (BSA), a representative serum protein, with moderate binding constants in a range of 10^3^–10^4^ M^−1^. The bindings of PF and CT did not induce noticeable BSA’s structural changes. Both pesticides bound preferentially to the site I pocket of BSA, where the hydrophobic interaction was the main binding mode of PF, and the electrostatic interaction drove the binding of CT. As a result, PF and CT may not only induce direct toxicity by themselves, but also compete with therapeutic drugs and essential substances to sit in the Sudlow site I of serum albumin, which may interfere with the pharmacokinetics and equilibrium of drugs and other substances causing consequent adverse effects.

## Introduction

Pesticides are extensively utilized worldwide to protect crops and households from invaders like pests, weeds, microbes, and insects. Also, pesticides are frequently used in public health and domestic settings, especially for controlling vector-borne diseases and prolonging the service life of many materials (e.g., leathers, plastics, and fabrics). Unfortunately, pesticides can adversely affect human health, as they cause acute poisoning and chronic health effects, such as sudden death, cancers, metabolic and endocrine dysfunctions, developmental disorders, reproductive problems, and neurological impairments. Pesticides generally enter human body via ingestion, inhalation, and skin absorption. Particularly, farmers and residents near application areas have potential risks of direct contact with pesticides of high doses and persistent exposures. General population seems to be exposed to low pesticide contamination via foods and drinks, resulting in a long-term accumulation of pesticide residues, and eventually causing a risk of chronic effects including cancer and other related diseases.

**Figure 1:**
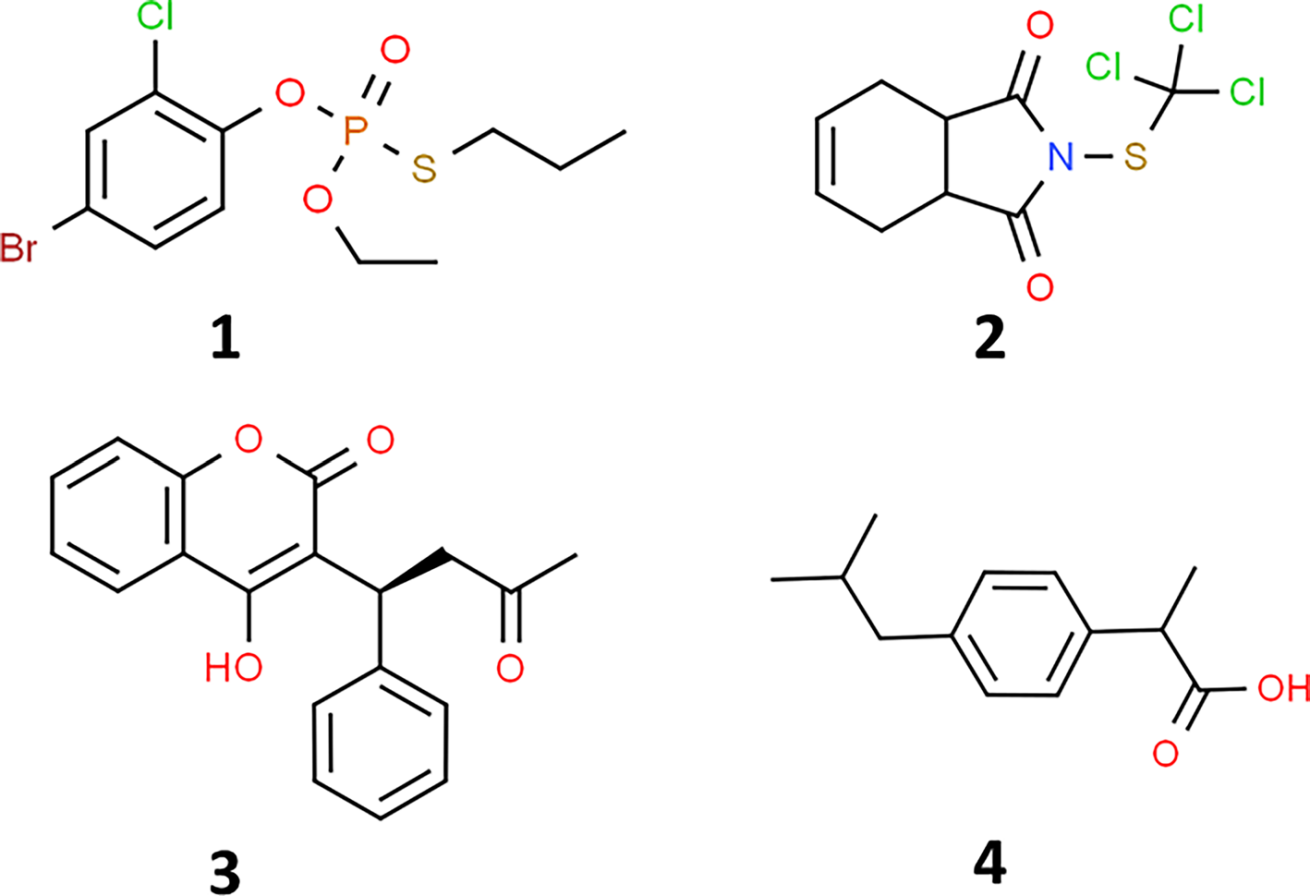
Chemical structures of profenofos (**1**), captan (**2**), warfarin (**3**), and ibuprofen (**4**).

After the pesticides enter the human body, their accumulation and toxicity greatly depend on the physicochemical and toxicokinetic properties, including absorption, distribution, metabolism, excretion, and toxicity (ADMET), which can be analogized to the pharmacokinetics of therapeutic drugs^1^. In general, exogenous substances, such as drugs, toxins, and vitamins, are transported via the bloodstream to the effective areas by serum protein carriers, in which albumin is the most abundant protein in the serum of humans and other mammals^2^. In addition to serving several physiological functions (e.g., maintaining oncotic pressure, controlling homeostasis, and providing antioxidative and anticoagulant effects), serum albumin also acts as a key player in regulating pharmacokinetics and pharmacodynamics of drugs and toxicants^3^. Serum albumin possesses a suitable conformation and surface characteristics to bind with a variety of chemical substances, making it a decent transporter of drugs and toxins toward the effective sites and to be metabolized in the hepatocellular system. Particularly, serum albumin is structurally conserved among mammals and consists of three domains (I, II, and III), where two drug binding sites, namely Sudlow’s site I and site II, have been identified on subdomains IIA and IIIA, respectively. Site I preferentially binds with bulky heterocyclic anions, while site II favors binding with aromatic carboxylic compounds via non-covalent bondings^4,5^. With the employment of multi-spectroscopic techniques, the interaction between serum albumins and ligands of interest has been extensively investigated for its toxicological profiles such as food dyes^6^, metallopharmaceuticals^7^, and photosensitizers^8^. Also, the influence of serum albumin on the blood concentration of pesticides has been widely reported. Compared to the unbound forms, the albumin-bound chemical species are more tolerant to degradation and excretion; thus, serum albumin plays a crucial function in controlling the half-life and actions of drugs and toxicants. A postmortem study revealed that malathion bound favorably with lysine and cysteinyl proline residues of human serum albumin (HSA), causing the decrease in free malathion in serum to be undetectable level^9^. Bindings of serum albumin with various organophosphorus insecticides, i.e., diisopropylfluorophosphate, chlorfenvinphos, chlorpyrifos, and diazinon, were reported by Tarhoni and colleagues^10^. In vitro investigation revealed that chlorpyrifos was able to bind with HSA at 3.039 × 10^5^ M^−1^ of a binding constant^11^. The mechanism for the binding of chlorpyrifos, parathion-methyl, and malathion with serum albumin mainly involved hydrophobic interaction^12^. The binding of HSA and carbamate pesticides was documented, and their binding parameters could be used to predict acute toxicity by means of an artificial neural network model^13^. In addition, serum albumin binding has been investigated for a number of pesticides, such as atrazine^14^, propanil and bromoxynil herbicides^15^, tebuconazole^16^ and iprodione^17^ fungicides, and carbofuran insecticide^18^. However, these efforts do not address the several hundred pesticides used worldwide; thus, this study aimed to explore in-depth interaction mechanisms of serum albumin with PF and CT (Fig. 1) as representatives of insecticide and fungicide classes that have been used worldwide, respectively.

**Figure 2:**
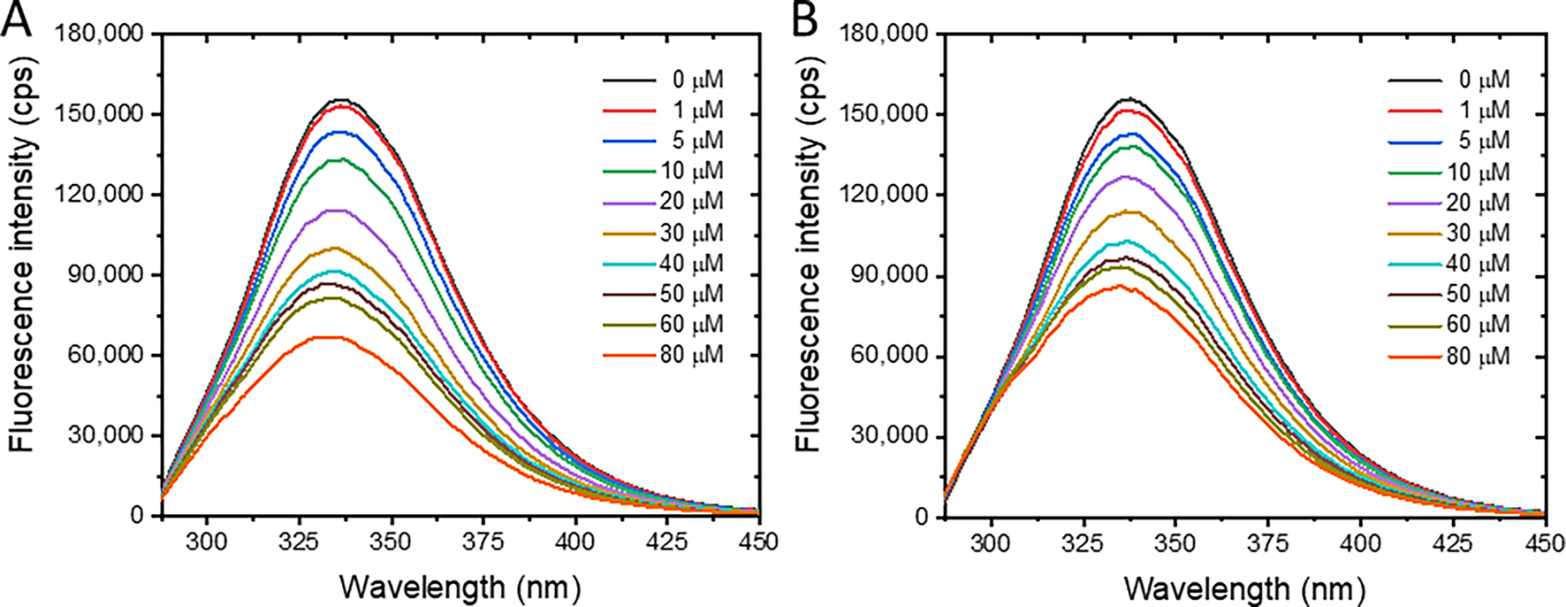
Fluorescence spectra of BSA in the presence of PF (**A**) or CT (**B**). Concentration of BSA was 2 μM, while pesticide concentrations were varied in a range of 0–80 μM at 310 K.

Profenofos (4-bromo-2-chloro-1-[ethoxy(propylsulfanyl)phosphoryl]oxybenzene; PF) is an organophosphorus insecticide (Fig. 1; **1**), but it contains an S-alkyl (S–C_3_H_7_) group attached to the phosphorus instead of a typical diethoxy (O–C_2_H_5_) group as other organophosphorus members^19^. PF is solely used for controlling insects and mites on cotton, maize, sugar beet, soya beans, potatoes, etc.^20^. It is extremely toxic to the nervous system by inhibiting acetylcholinesterase (AChE) in the synaptic clefts of neuromuscular junctions via the phosphorylation of serine residue at the enzyme’s active site^21^. Captan (2-(trichloromethylsulfanyl)-3a,4,7,7a-tetrahydroisoindole-1,3-dione; CT) is a phthalimide class of fungicides (Fig. 1; **2**), that is widely used in agriculture and serves as a preservative in pharmaceuticals, cosmetics, textiles, plasticizers, and paints. It produces acute effects, including vomiting, diarrhea, dermatitis, and conjunctivitis, and long-term exposure to high doses can cause cytotoxicity and regenerative cell hyperplasia; thus, CT is considered to be a potential carcinogen^22^. Accordingly, the interaction between serum albumin and CT or PF can cause important implications on homeostasis and several regulatory processes of the human body, so further investigation is still required to determine the full extent of interaction processes and the potential consequences on human health. Therefore, this study utilized various biophysical and computational analyses to explore mechanistic interactions between serum albumin and either PF or CT, which include ADMET predictions, multi-spectroscopic approaches (e.g., UV–Vis absorption, fluorescence, and circular dichroism spectroscopy), mathematical model analyses, molecular dockings, and molecular dynamics simulations.

## Results and discussion

### Toxicokinetic prediction

Toxicokinetic properties analogous to the pharmacokinetics of PF and CT were predicted in silico using SwissADME, pkCSM, and ProTox-II tools, and the estimated ADMET properties are summarized in Table 1. PF and CT are small molecular structures with molecular weights (MW) of 373.63 and 300.59 g/mol, respectively, that have comparable topological polar surface areas. Both pesticides are likely lipophilic, belonging to the halo arene and alkyl triphosphate groups of PF and the trichloromethyl derivative of isoindole structure of CT. As a result, they likely accumulate in lipid compartments of the human body. The predicted results showed that PF and CT exhibit high absorptivity through the gastrointestinal tract and derma; thus, oral ingestion and skin contact should be the main routes by which these pesticides enter the body. The predicted log BB values of PF and CT were 0.279 and 0.307, respectively, which were concurrent with the predicted CNS permeability indices (log PS) and indicated that they can pass through the blood–brain barrier toward the central nervous system^23,24^. The predicted toxicities of PF and CT were comparable with the reports of the U.S. Environmental Protection Agency (US EPA)^21,25^. PF is an organophosphorus pesticide and has been proven to be a neurotoxic agent by inhibiting AChE^26^. Previous studies reported that AChE and toxicologically active metabolite, 4-bromo-2-chlorophenol, biomarkers could be monitored in both blood and urine of non-occupational and occupational exposures to PF in Egyptian cotton fields^27,28^. Similarly, key metabolite tetrahydrophthalimide of CT was also detected in urine and plasma of dermally and orally exposed workers^29,30^. These toxicokinetic predictions of PF and CT were along with the initial breakdown metabolites in human circulation. Moreover, the 50% lethal doses (LD_50_) via the oral route were reported to be in a range of 260–800 mg/kg, which were comparable with the predicted LD_50_ (162 mg/kg) in this study. PF is classified as a “Group E” chemical, as evidenced by its lack of carcinogenicity^21^. Besides, PF was predicted to be inactive for other toxicities as listed in Table 1. Even if CT has a high predicted LD_50_ at 7000 mg/kg, it was estimated to be a carcinogen and mutagen. Nevertheless, the US EPA has re-classified CT from a “Group B2” substance (probable human carcinogen) to be “not likely carcinogen” since 2004^25^, in which this contradiction may be due to the inadequate updating of the prediction database.

**Table 1:** ADMET properties of PF and CT obtained from SwissADME, pkCSM, and ProTox-II.

ADMET	PF	CT
**Physicochemical properties**		
MW (g/mol)	373.63	300.59
Number of hydrogen bond acceptors	3	2
Number of hydrogen bond donors	0	0
Topological polar surface area (Å^2^)	70.64	62.68
Water solubility	− 5.766	− 3.965
Octanol/water partition coefficient (log P_o/w_)	4.20	2.18
**Absorption**		
GI absorption (% absorbed)	High (87.367)	High (93.998)
Skin permeability (log Kp)	High (− 2.862)	High (− 2.214)
**Distribution**		
Fraction unbound (Fu)	0.101	0.475
BBB permeability (log BB)	Yes (0.279)	Yes (0.307)
CNS permeability (log PS)	− 2.343	− 2.865
**Metabolism**		
CYP2D6 substrate	No	No
CYP3A4 substrate	No	No
**Excretion**		
Total clearance (log mL/min/kg)	0.147	0.135
Renal OCT2 substrate	No	No
**Toxicity**		
Hepatotoxicity	Inactive	Inactive
Carcinogenicity	Inactive	Active
Immunotoxicity	Inactive	Inactive
Mutagenicity	Inactive	Active
Cytotoxicity	Inactive	Inactive
Skin sensitization	Inactive	Active
Predicted LD_50_ (mg/kg)	162	7000

### Fluorescence quenching of BSA by pesticides

BSA exhibited a distinct fluorescence maximum at 340 nm when excited at a wavelength of 280 nm, exhibiting the intrinsic characteristics of amino acids containing aromatic groups, i.e., tryptophan (Trp), tyrosine (Tyr), and phenylalanine (Phe). Both PF and CT pesticides dramatically quenched the fluorescence emission of BSA in a concentration-dependent fashion; however, PF exerted a slightly higher quenching effect than that of CT (Fig. 2A,B). Neither peak shift nor extra peak production was observed. Fluorescence loss of BSA can occur by many mechanisms, especially structural changes, complex formation, and denaturation because of ligand exposure^31^, suggesting that the interaction phenomenon between BSA and PF or CT was taken place in this circumstance.

**Figure 3:**
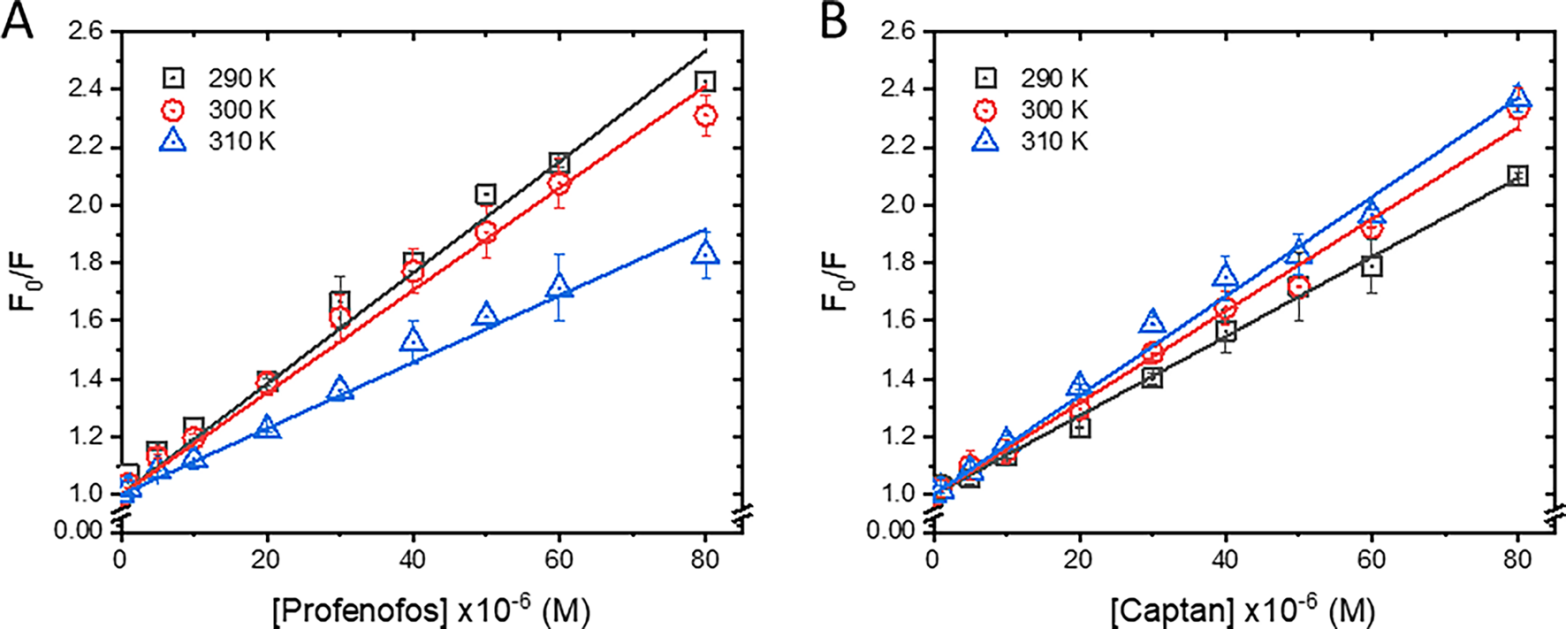
Stern–Volmer plots of BSA–PF (**A**) and BSA–CT (**B**) at 290, 300, and 310 K.

To determine the interaction mechanisms between BSA and pesticides, the Stern–Volmer model (Eq. (1)) was applied to the fluorescence data of the BSA–PF and BSA–CT reactions. The relative fluorescence of BSA in the absence (*F*_0_) and presence (*F*) of pesticide was plotted against the relevant pesticide concentration (*Q*) as shown in Fig. 3.

1 $$\frac{{F_{0} }}{F} = 1 + K_{sv} \left[ Q \right] = 1 + k_{q} \tau_{0} \left[ Q \right]$$

*F*_0_ and *F* are the fluorescence intensities at 340 nm emission wavelength of BSA in the absence and presence of pesticide, respectively. $K_{sv}$ is the Stern–Volmer constant, and $\left[ Q \right]$ is the pesticide concentration. $k_{q}$ and $\tau_{0}$ denote the quenching rate constant and fluorescence lifetime of unquenched BSA (6.2 ns)^32^, respectively.

The Stern–Volmer plots were well-fitted with a linear function, indicating that the interaction between BSA and PF (Fig. 3A) or CT (Fig. 3B) was mostly driven by a single mechanism, either static or dynamic quenching^33^. The static process occurs via the complex formation between the quencher (pesticide) and fluorophore (BSA) at the ground-state level, whereas the dynamic quenching involves the collision between BSA and pesticide molecule under excited-state conditions. Typically, static and dynamic processes can be differentiated by determining the $K_{sv}$ values or the slopes of Stern–Volmer plots derived from different reaction temperatures^34^. Theoretically, rising the temperature of reaction enhances the motion and collision of molecules; as a result, the $K_{sv}$ value of the dynamic quenching process increases. The static process is affected by the rising temperature in vice versa because high temperature lessens the complex formation between protein and ligand as indicated by the reduction of $K_{sv}$ value. In this study, Stern–Volmer plots of BSA–PF and BSA–CT were determined at the following different temperatures to cover a wide temperature range from hypothermic (290 and 300 K) to physiological (310 K) conditions (Fig. 3). The $K_{sv}$ values of BSA–PF decreased with increasing temperatures (Fig. 3A and Table 2), suggesting that the BSA and PF interaction is driven by the static quenching process. On the contrary, $K_{sv}$ values of the BSA‒CT system slightly increased with the rising temperature (Fig. 3B), which inferred that the dynamic quenching process is also involved in the interaction. However, its quenching rate constants ($k_{q}$) was at a level of 10^12^ M^−1^ s^−1^, which was 100 times larger than the maximum diffusion rate (2 × 10^10^ M^−1^ s^−1^) of the dynamic quenching process. This contradicted observation may suggest that complex formation plays a more significant role in the interaction between BSA and CT, rather than a dynamic process being the only driving factor, which was concurrent with a static mechanism of phthalimide analogs forming a stable complex with BSA^35^. However, further investigation is necessary to confirm this hypothesis.

**Table 2:** Stern–Volmer and quenching rate constants of BSA‒PF and BSA‒CT.

System	*T* (K)	$K_{sv}$ × 10^4^ (M^−1^)	$k_{q}$ × 10^12^ (M^−1^ s^−1^)	r^2^
BSA‒PF	290	1.92 ± 0.05	3.09 ± 0.08	0.9986
	300	1.77 ± 0.04	2.85 ± 0.07	0.9989
	310	1.15 ± 0.05	1.85 ± 0.05	0.9990
BSA‒CT	290	1.37 ± 0.02	2.23 ± 0.03	0.9997
	300	1.59 ± 0.03	2.58 ± 0.05	0.9997
	310	1.71 ± 0.03	2.71 ± 0.05	0.9993

### UV–Vis absorption spectra

**Figure 4:**
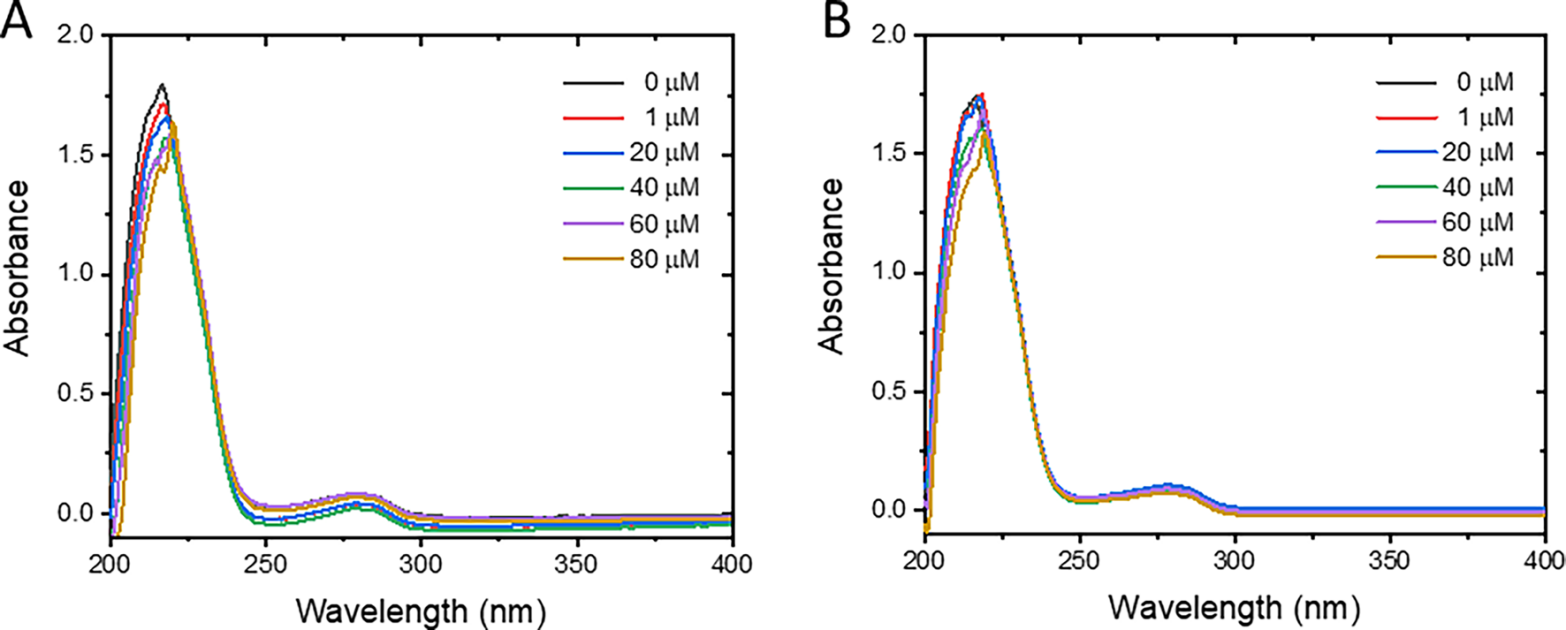
UV–Vis absorption spectra of BSA in the presence and absence of PF (**A**) and CT (**B**) after subtracting the intrinsic characteristics of pesticides. Original absorption spectra of pesticides in the presence and absence of BSA are presented in the supplementary information (Fig. S1).

UV–Vis absorption spectroscopy is a valuable technique for detecting complex formation and dynamic collision between protein and ligand^36^. Complex formation is a static process that occurs at the ground state and can affect the electronic properties of the host protein. This event can be observed through the changes in the UV–Vis absorption^31^. However, in case the dynamic quenching process plays a major role, no changes in the absorption spectra should be observed because the interaction occurs at the excited state of the fluorophore. Herein, BSA exhibited typical absorption characteristics with two prominent peaks at ~ 217 and ~ 278 nm (Fig. 4 and Fig. S1), belonging to the properties of polypeptide backbone and aromatic amino residues, respectively. PF and CT exhibited small intrinsic absorption bands in the range of 200–250 nm, which could overlap with the polypeptide band of BSA (Fig. S1). These bands need to be subtracted from the BSA-pesticide systems, as illustrated in Fig. 4. Once exposed to either PF or CT, the backbone absorption peak was decreased and slightly shifted to the right by 3 nm (from ~ 217 to ~ 220 nm) by 80 µM PF or CT, indicating the structural perturbation of BSA by the pesticide. A typical absorption peak of Trp, Phe, and/or Tyr residues of BSA at ~ 278 nm remained unchanged in the presence of CT, while it was faintly enhanced by PF without any peak shift. These observations suggested that both PF and CT can bind to BSA at the ground-state level, which deforms the microenvironment of BSA’s backbone but does not vastly affect the aromatic amino residues.

### Two-dimensional conformations of BSA against pesticide exposure

**Figure 5:**
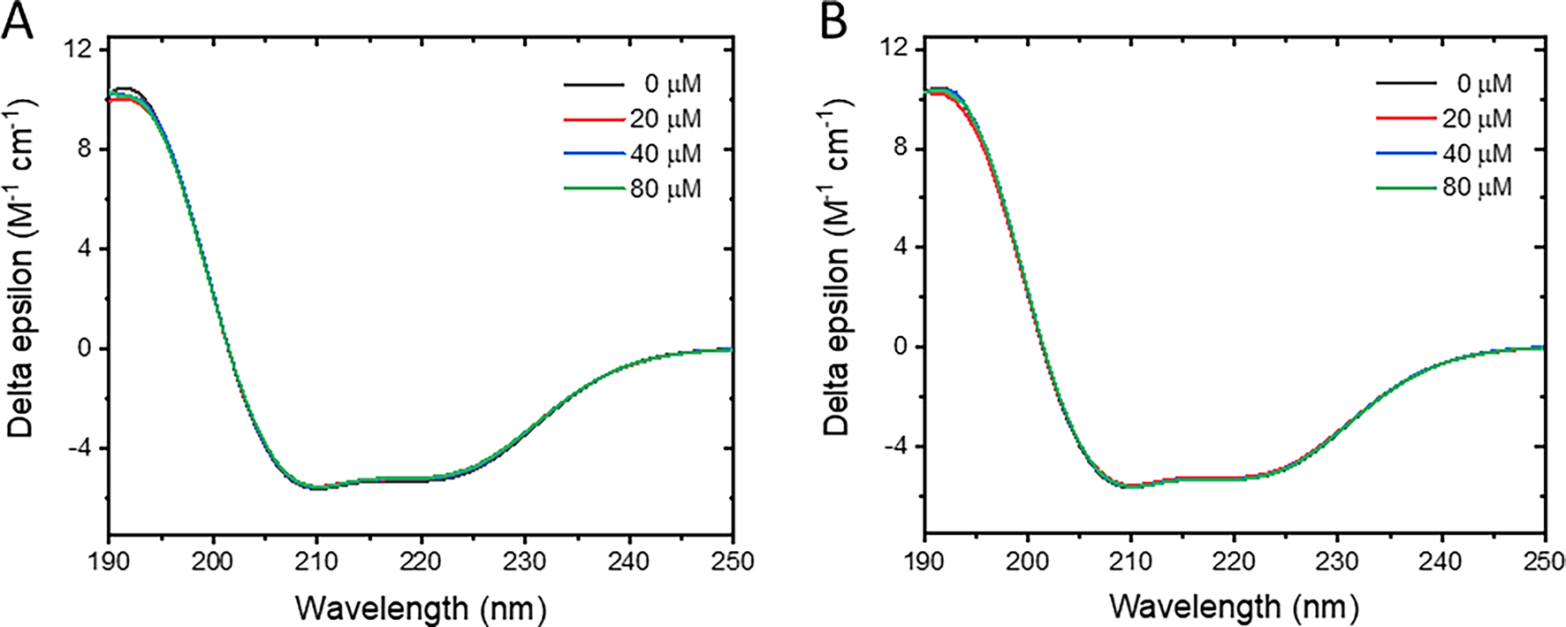
Circular dichroism (CD) spectra of BSA in the presence and absence of PF (**A**) and CT (**B**). Concentration of BSA was 2 µM, and the pesticide concentrations were 20, 40, and 80 µM.

Circular dichroism (CD) spectroscopy was performed to elucidate the effect of PF and CT on structural stability of BSA. A typical CD spectrum of the α-helix conformation was observed on pure BSA, in which two negative bands at ~ 208 and ~ 222 nm were prominent^37^, and its α-helix content was estimated to be 44.7% (Fig. 5, Table S1). Upon exposed BSA with either PF or CT at 20, 40, and 80 μM, the substantial changes in CD spectra were unable to distinguish, which led to the conclusion that the binding of PF or CT does not drastically destabilize the BSA structure.

### Binding affinity and thermodynamics of BSA and pesticide interaction

**Figure 6:**
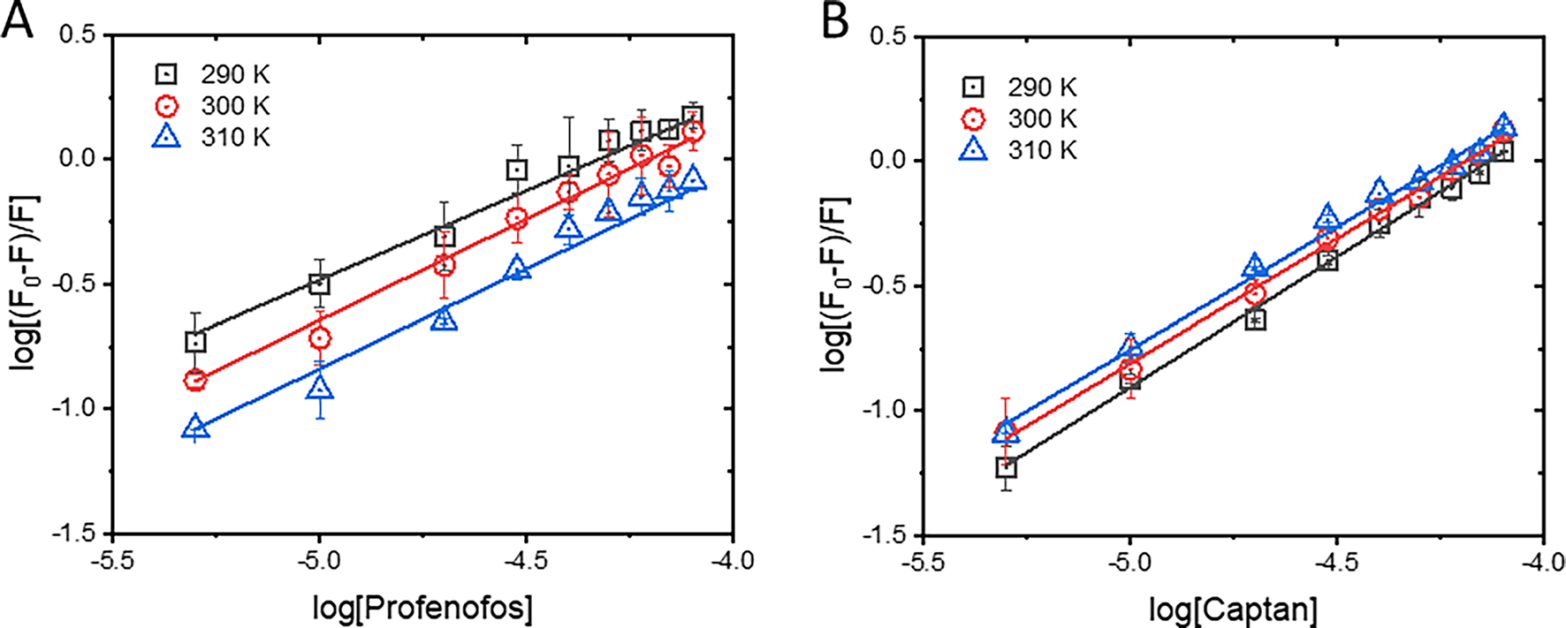
Double-logarithmic plots of BSA–PF (**A**) and BSA–CT (**B**) at 290, 300, and 310 K. Concentration of BSA was a fixed constant at 2 μM, while varying concentrations of PF or CT were in a range of 0–80 μM.

In this study, the double-logarithmic plot has been applied to elucidate the binding parameters of BSA–PF and BSA–CT complexes that can reflect both the binding constant together with the cooperativity or stoichiometry of the binding event^6^. The affinities between BSA and PF or CT were determined by plotting the concentrations of PF and CT against the relative fluorescence changes of unbound and pesticide‒bound BSA in the logarithmic term (Fig. 6) according to Eq. (2).

2 $$log\frac{{(F_{0} - F)}}{F} = logK_{a} + nlog\left[ Q \right]$$

*F*_0_ and *F* are the fluorescence intensities of BSA without and with pesticide, respectively. $Q$ is the molar concentration of the pesticide. $K_{a}$ and $n$ are the binding constant and the number of binding sites of pesticide on BSA, respectively.

The double-logarithmic plots of BSA–PF and BSA–CT at different temperatures were well-fitted with a linear model of Eq. (2), in which the obtained $K_{a}$ and $n$ are shown in Table 3. Both PF and CT exhibited low to moderate affinity toward BSA, and the binding constants fell within the range of 10^3^–10^4^ M^−1^ at temperatures between 290 and 310 K and showed the number of binding site parameter in a range of 0.857–1.15, indicating one binding site without cooperative binding of the pesticide on BSA molecule^8^. The affinity of BSA–PF complex was about 10 times weaker than the BSA–CT complex. These weak binding affinities of both pesticides can modify their release from BSA binding pocket, leading to an elevated concentration of the free form and causing harmful toxicity. Increasing the temperature enhanced the binding affinity between BSA and PF, suggesting that the complex formation was mainly driven by hydrophobic interactions^38^. However, the temperature caused a different effect on the BSA–CT complex, in which its binding constant was decreased as the temperature increased, indicating the involvement of hydrogen bonding and electrostatic driven the BSA and CT interaction.

**Table 3:** Binding constants, numbers of binding sites, and thermodynamic parameters of BSA–PF and BSA–CT.

System	*T* (K)	$K_{a}$ (M^−1^)	*n*	r^2^	$\Delta G^{0}$ (kJ mol^−1^)	$\Delta S^{0}$ (J mol^−1^ K^−1^)	$\Delta H^{0}$ (kJ mol^−1^)
BSA–PF	290	2354 ± 1.48	0.77 ± 0.04	0.9839	− 18.81		
	300	3425 ± 1.46	0.84 ± 0.04	0.9873	− 20.11	130.14	18.93
	310	3897 ± 1.49	0.89 ± 0.04	0.9874	− 21.41		
BSA–CT	290	20,741 ± 1.30	1.04 ± 0.03	0.9960	− 23.65		
	300	15,523 ± 1.26	1.00 ± 0.02	0.9966	− 24.01	35.84	− 13.26
	310	14,581 ± 1.40	0.98 ± 0.03	0.9926	− 24.37		

To gain further insight into the binding mechanism of the BSA–PF and BSA–CT complexes, thermodynamic parameters were investigated (Table 3) using Eqs. (3) and (4). Gibbs free energy ($\Delta G^{0}$) values of both BSA–PF and BSA–CT were negative, indicating that complex formations were spontaneous processes. In addition, the signs of enthalpy ($\Delta H^{0}$) and entropy ($\Delta S^{0}$) can provide information about the types of intermolecular forces involved in the complex formation. When $\Delta H^{0}$ and $\Delta S^{0}$ are both negative, hydrogen bonding and van der Waals forces are likely involved. When $\Delta H^{0}$ is negative and $\Delta S^{0}$ is positive, electrostatic and ionic interactions are possible. Positive $\Delta H^{0}$ and $\Delta S^{0}$ suggest hydrophobic interactions, while positive $\Delta H^{0}$ and negative $\Delta S^{0}$ infer the non-spontaneous reaction. Herein, both $\Delta H^{0}$ and $\Delta S^{0}$ of BSA–PF were positive, which implied that hydrophobic interaction was the main binding mode. This finding showed well-agreement with the previous reports on the interaction of serum albumin with other organophosphorus insecticides (e.g., chlorpyrifos, malathion, parathion, and isocarbophos)^11,12,39^. However, electrostatic and ionic interactions should be the main binding force of the BSA‒CT complex, because its $\Delta H^{0}$ and $\Delta S^{0}$ were negative and positive, respectively.

3 $$\Delta G^{0} = \Delta H^{0} - T\Delta S^{0}$$

4 $$lnK_{a} = - \frac{{\Delta H^{0} }}{RT} + \frac{{\Delta S^{0} }}{R}$$

where $\Delta G^{0}$, $\Delta H^{0}$, and $\Delta S^{0}$ are the Gibbs free energy, enthalpy, and entropy, respectively. *R* is a gas constant (8.314 J mol^−1^ K^−1^). $K_{a}$ is the binding constant of the complex at a temperature $T$ K.

### Microenvironmental changes around tryptophan and tyrosine residues

BSA contains 18 Tyr residues along its polypeptide chain, and only two Trp residues are located at subdomain IB (Trp134) and subdomain IIA (Trp213). Analysis of the fluorescence characteristics of Tyr and Trp is valuable for interpreting the structural changes in protein–ligand complexes, as sensitive aromatic amino acids to microenvironmental changes. In many cases, ligand binding can alter the peptide backbone, leading to the exposure of Tyr and Trp residues to the surrounding aqueous solution and external milieu. Therefore, monitoring the fluorescence characteristics of Tyr and Trp can help reveal the structural changes in BSA that may occur after binding with PF or CT. Synchronous fluorescence spectroscopy is a simple and effective technique for determining the microenvironmental changes nearby the Trp and Tyr amino acids of the protein, in which the emission and excitation wavelengths are simultaneously scanned with constant offsets of 15 and 60 nm for visualizing characteristic emissions of Tyr and Trp, respectively^40,41^.

**Figure 7:**
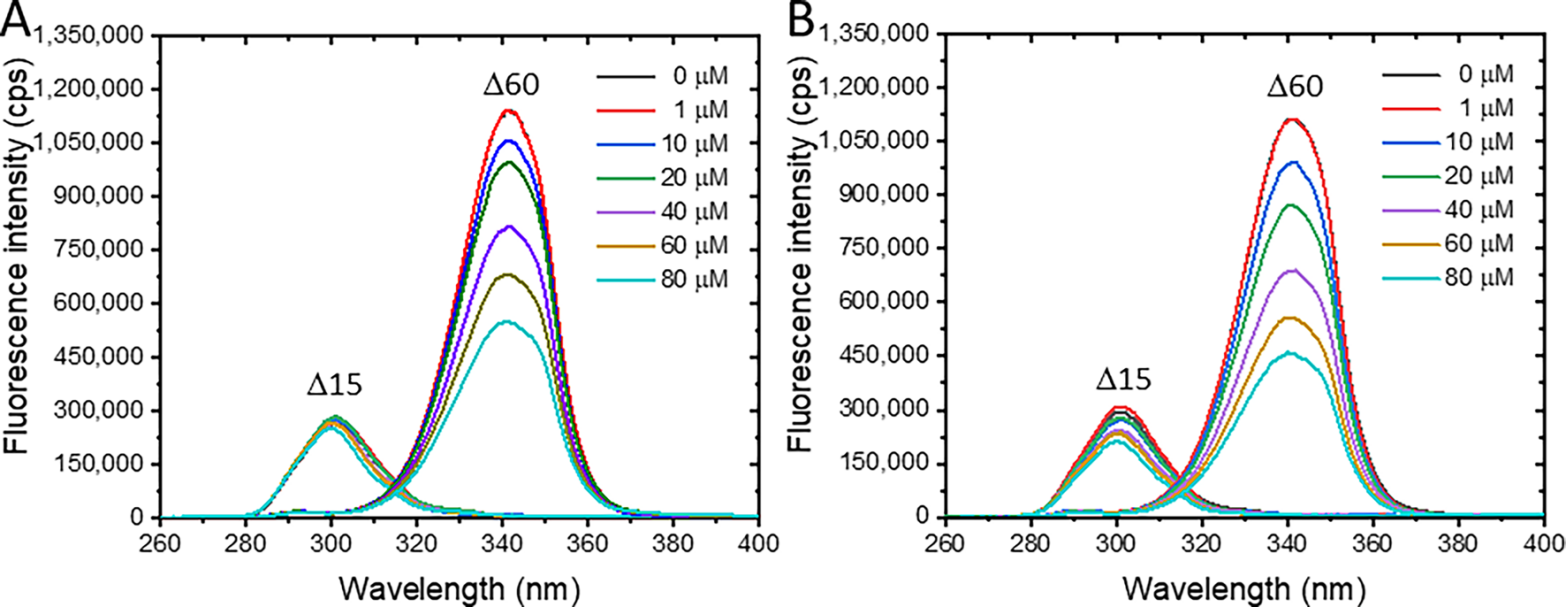
Synchronous fluorescence spectra of BSA in the presence of PF (**A**) and CT (**B**). Fluorescence characteristics of Tyr and Trp residues are labeled with Δ15 and Δ60, respectively.

The maximum emission peaks of Tyr and Trp in BSA occurred at 301 nm and 342 nm, respectively (Fig. 7). The fluorescence intensities of both Tyr and Trp were reduced with increasing concentrations of PF or CT. However, as compared to Tyr, Trp was found to be more sensitive to PF or CT exposure. In the presence of PF, a slight blueshift of approximately 2 nm was observed in both Tyr and Trp emission peaks at 80 μM PF (Fig. 7A). This result suggested that the Tyr and Trp residues may be exposed to a hydrophobic environment, possibly due to the non-polar properties of PF’s 4-bromo-2-chlorophenyl and O-ethyl S-propyl phosphorothioate structures. When exposed to CT, the fluorescence peak of Tyr exhibited a 1 nm blueshift, while the peak of Trp remained unchanged (Fig. 7B), indicating that the Tyr residue may be exposed to CT, while Trp remains buried inside the protein domain and do not directly contact with CT.

### Fluorescence resonance energy transfer (FRET) between BSA and pesticide

Protein fluorescence can be quenched not only by conformational changes but also by FRET. This process involves the transfer of energy from the protein's fluorophores (donors), such as Trp and Tyr, to neighboring acceptor molecules, i.e., PF and CT in this study, through dipole–dipole interactions. However, FRET can only occur if certain conditions meet the criteria, including (1) acceptor molecules locate within 2‒8 nm of the donor, (2) the fluorescence spectrum of the donor partially overlaps with the absorption spectrum of the acceptor, and (3) the transition dipoles of the donor and acceptor properly align with a parallel orientation being the most efficient for energy transfer. The FRET phenomenon can be determined using Eqs. (5)–(7).

5 $$E = 1 - \frac{F}{{F_{0} }} = \frac{{R_{0}^{6} }}{{R_{0}^{6} + r^{6} }}$$

6 $$R_{0} = 0.211\left\lfloor {\frac{{\kappa^{2} \emptyset_{D} J}}{{\eta^{4} }}} \right\rfloor^{\frac{1}{6}}$$

7 $$J = \frac{{\mathop \smallint \nolimits_{i}^{j } F\left( \lambda \right)\varepsilon \left( \lambda \right)\lambda^{4} d\lambda }}{{\mathop \smallint \nolimits_{i}^{j} F\left( \lambda \right)d\lambda }}$$

*E* is the efficiency of FRET. *F*_0_ and *F* denote the fluorescence intensities of BSA in the absence and presence of pesticide, respectively. *r* is the actual distance between the donor and acceptor. Förster distance (*R*_0_) is the distance, which half of the excitation energy is transferred to the acceptor by using Eqs. (6) and (7). $\kappa^{2}$ is the orientation factor between donor and acceptor. $\emptyset_{D}$ and $\eta$ are the fluorescence quantum yield of the donor and the refractive index of the medium, estimated to be 0.15 and 0.3139, respectively in these systems. *J* is the spectral overlap integral of the donor’s fluorescence spectrum and the acceptor’s absorption spectrum in a wavelength (λ) range of *i* (285 nm) and *j* (450 nm) according to Eq. (7).

**Figure 8:**
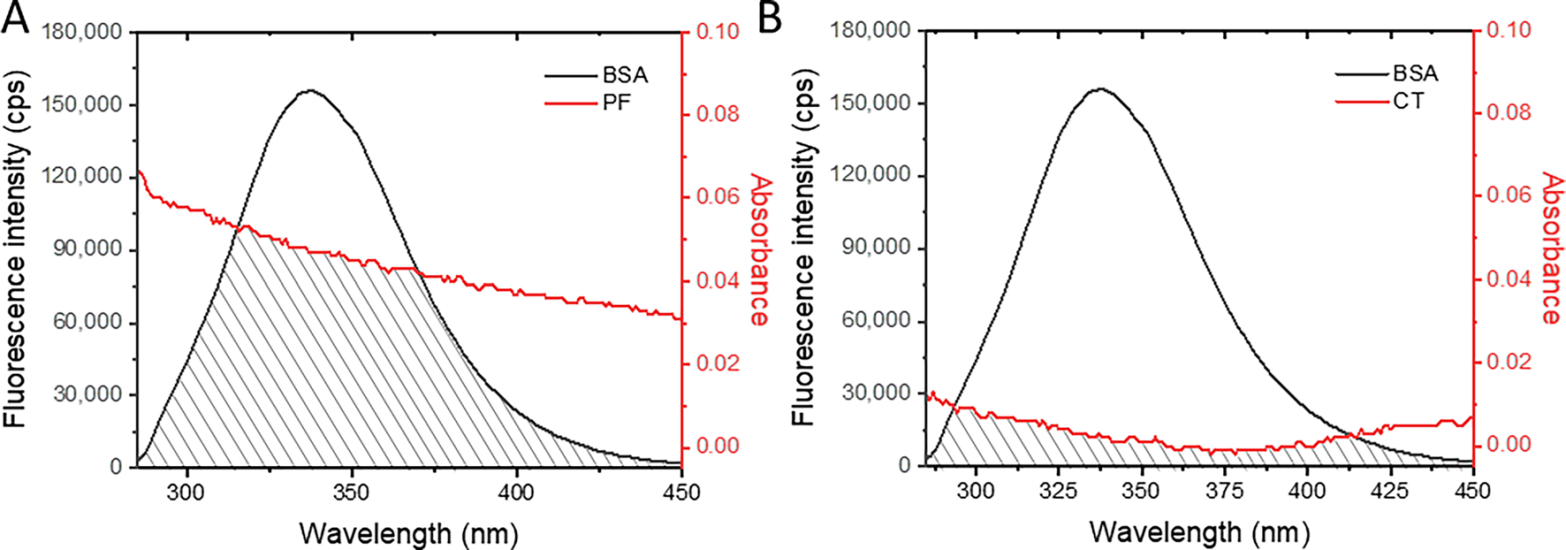
Spectral overlaps (shaded areas) of BSA’s fluorescence spectrum (black curve) and PF’s (**A**) or CT’s (**B**) absorption spectrum (red curve). [BSA] = 2 μM, [PF] or [CT] = 5 μM.

Herein, the overlapping spectra of BSA’s fluorescence and absorbance of PF and CT are shown in Fig. 8A,B, respectively. FRET efficiencies between BSA (2 µM) and either PF or CT (5 µM) were approximately similar at 0.077 and 0.076, respectively. However, the integral spectral overlap of BSA‒PF was about 100 times higher than that of the BSA‒CT because the PF could absorb more photons in the BSA’s fluorescence region (Table 4). Based on the assumption that the transition dipoles of BSA’s fluorophore and pesticide were parallel orientation, the $\kappa^{2}$ factor of the system was estimated to be 0.476. The actual distances of BSA’s fluorophore were 3.83 nm to PF and 2.25 nm to CT. These calculated distances were in a range of 0.5 *R*_0_ to 1.5* R*_0_ and less than 10 nm, which suggested that fluorescence quenching of BSA by PF or CT can occur via the FRET phenomenon, thus implying the complex formation between BSA and PF or CT.

**Table 4:** FRET parameters of BSA‒PF and BSA‒CT.

System	$E$	$J$ (M^−1^ cm^−1^ nm^4^)	$R_{0}$ (nm)	$r$ (nm)	0.5 $R_{0}$ < $r$ < 1.5 $R_{0}$
BSA‒PF	0.077	1.33 × 10^14^	2.53	3.83	Yes
BSA‒CT	0.076	5.35 × 10^12^	1.48	2.25	Yes

### Competitive binding of BSA and pesticide with site probes

Serum albumin plays a critical role in transporting various molecules, including endogenous compounds (e.g., fatty acids, hormones, and vitamins) and exogenous molecules (e.g., toxins and therapeutic drugs) to different parts of the body. There are two primary binding sites on serum albumin, known as site I and site II, that are responsible for binding to a wide range of ligands, especially aromatic and heterocyclic compounds. Site I is in the hydrophobic cavity of the subdomain IIA and can accommodate larger ligands, while site II is located near the surface of the protein at subdomain IIIA and can bind to smaller ligands. Thus, when the binding site of PF and CT on BSA is identified, the information provides valuable insights into their toxicities, drug interactions, and environmental impacts. These can inform the development of safer and more effective drugs, as well as environmental management strategies. A competitive displacement study with site probes can indicate the binding site of PF or CT on BSA, in which the commonly used site probes for site I and site II are warfarin and ibuprofen, respectively (Fig. 1). If a pesticide binds to the same pocket as a specific site probe on BSA, the binding constant of the BSA‒pesticide complex may be altered in the presence of site probe. The decreasing affinity indicates competitive binding, while the increasing affinity suggests cooperative binding between the pesticide and the site probe on BSA. In the presence of 2 µM of either warfarin or ibuprofen, the binding constants of both BSA‒PF and BSA‒CT were significantly reduced (as shown in Table 5, Fig. S2), suggesting that PF and CT may bind to BSA at or near the site I and site II pockets. However, PF preferentially binds at site I of BSA, as evidenced by a 23-fold decrease in binding constant upon co-incubation with warfarin, compared to a six-fold reduction observed in other competitive scenarios.

**Table 5:** Binding constants and binding site numbers of BSA‒PF and BSA‒CT.

System	Competitor	$K_{a}$ (M^−1^)	n	r^2^
BSA‒PF	Without	3897 ± 1.49	0.89 ± 0.04	0.9874
	Warfarin	172 ± 1.65	0.63 ± 0.03	0.9894
	Ibuprofen	669 ± 1.51	0.73 ± 0.02	0.9862
BSA‒CT	Without	14,581 ± 1.39	0.98 ± 0.03	0.9926
	Warfarin	4106 ± 1.49	0.89 ± 0.04	0.9870
	Ibuprofen	2331 ± 1.37	1.03 ± 0.03	0.9939

### Molecular docking simulation of BSA‒pesticide complex

**Figure 9:**
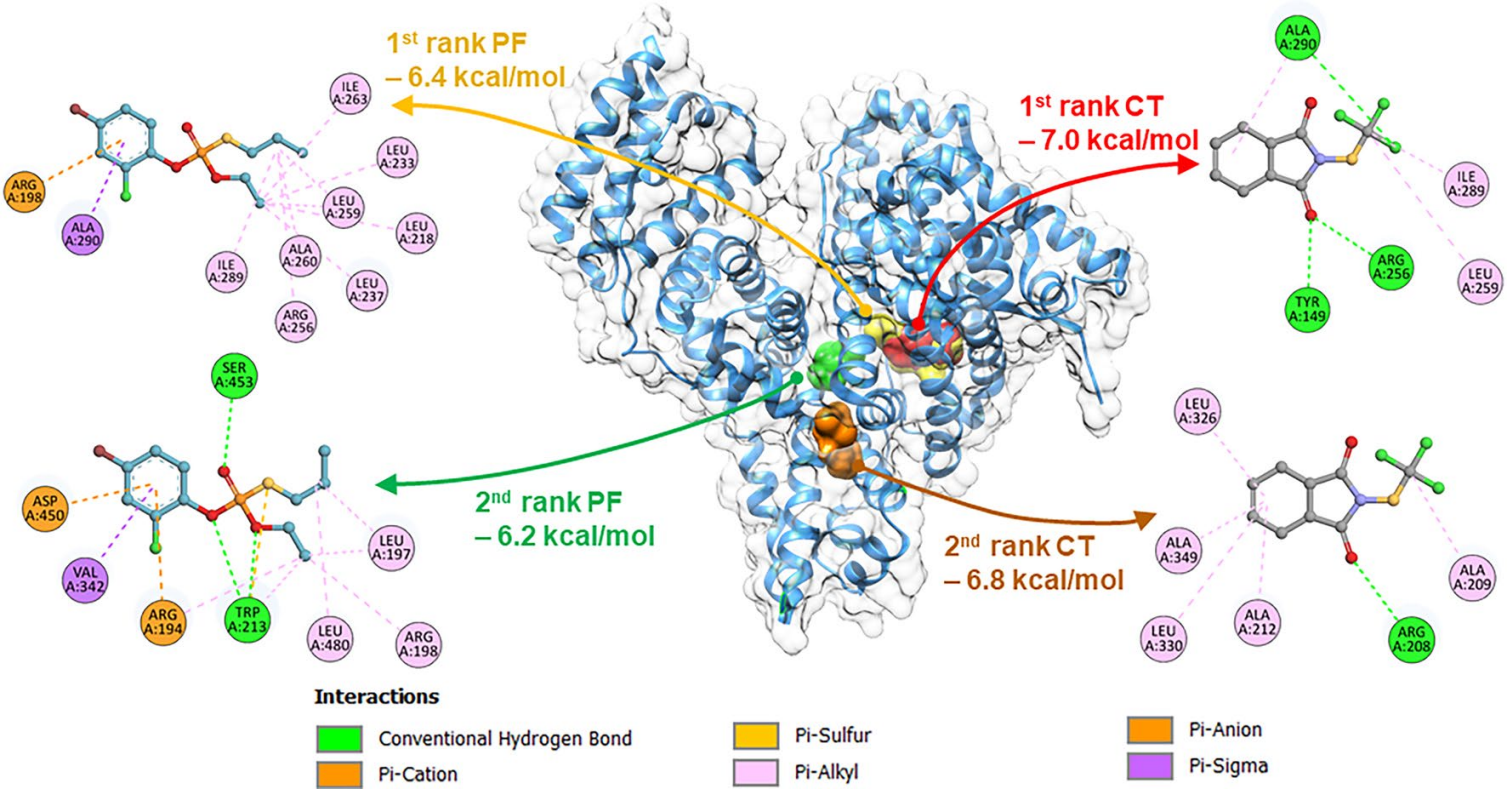
Preferable binding sites, relevant binding energies, and binding networks of PF and CT on the BSA structure obtained from AutoDock Vina.

**Figure 10:**
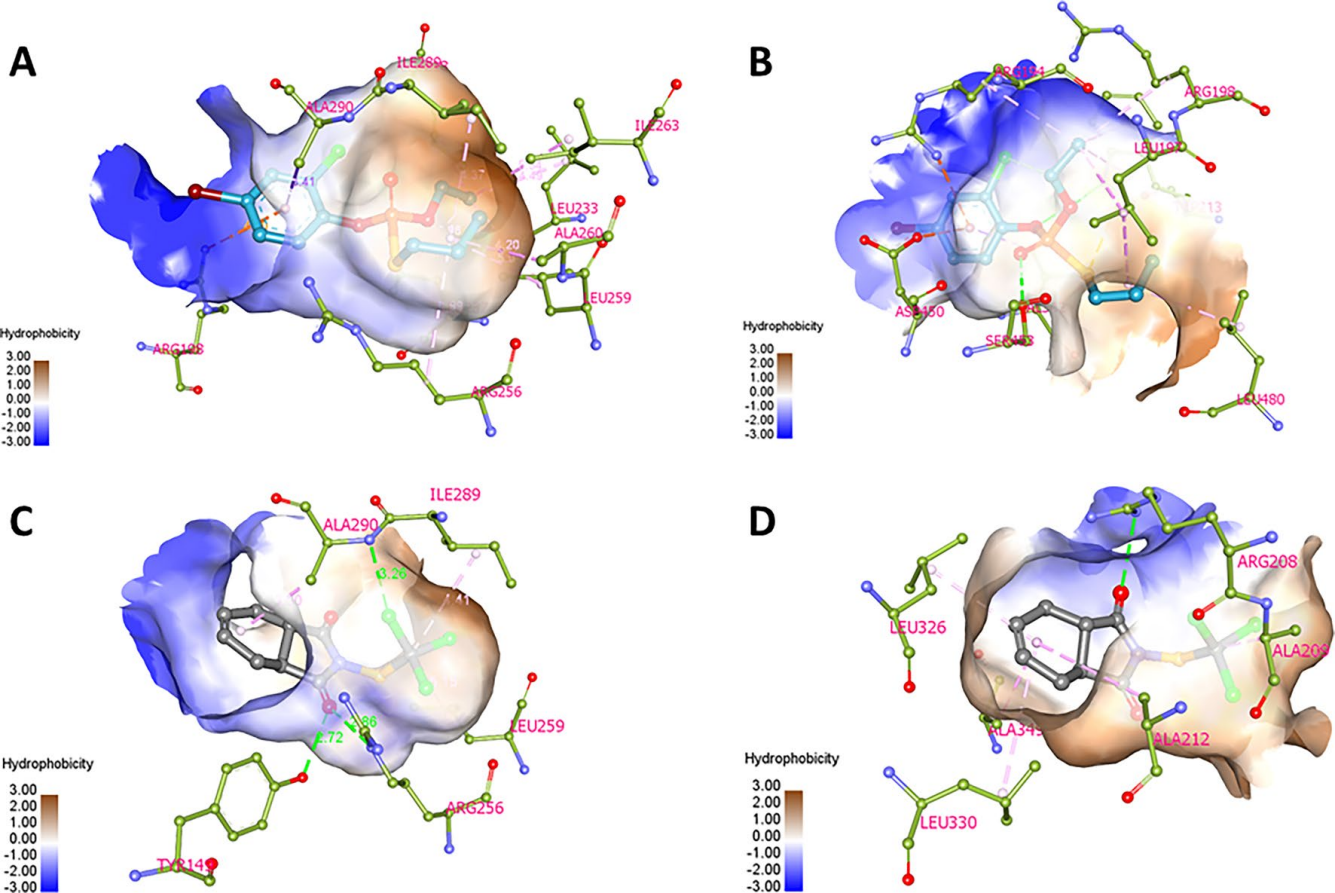
Binding pockets and interaction networks of BSA residues with the 1st (**A**,**C**) and 2nd (**B**,**D**) rank docked poses of PF (**A**,**B**) and CT (**C**,**D**).

To further investigate the interaction between BSA and either PF or CT pesticide, molecular docking analyses were performed using AutoDock Vina. The results of the docking simulations indicated that both PF and CT bind favorably to a specific region of the BSA structure known as the site I pocket (Fig. 9). The calculated binding energies of PF and CT were − 6.4 and − 7.0 kcal/mol, respectively. In addition to the site I pocket, other potential binding sites for PF and CT were identified as the second most preferable sites located in the lower area of the protein cleft (PC_lower_) between subdomains IIA and IIIA of BSA. However, PF and CT bind to PC_lower_ in different coordination modes. The calculated binding energies of PF and CT at PC_lower_ were − 6.2 and − 6.8 kcal/mol, respectively. These findings were consistent with other investigations, such as FRET, thermodynamics, and competitive binding analyses, which helped verify that the binding sites of PF and CT are at or near the site I and site II binding pocket of BSA. PF mainly exerted hydrophobic interaction with Leu, Ala, Ile, and Arg residues via its alkyl phosphorothioate moieties, while CT employed its tetrahydrophthalimide to form hydrogen bonds with Ala, Tyr, and Arg residues in the site I pocket of BSA (Figs. 9 and 10), which perfectly coincided with the findings of thermodynamic analysis. Furthermore, Trp212 of BSA is located close to the site I, where the FRET phenomenon could easily occur as the aforementioned. Therefore, site I of BSA should be the predominant binding site of PF and CT. Moreover, the PC_lower_ region is considerably bulky serving as a binding site for various molecules, such as hydroxytyrosol^42^, 5-amino-8-hydroxyquinoline^43^, and ibuprofen^44^. Also, the PC_lower_ region of BSA could potentially serve as a binding pocket for PF and CT, particularly under saturated conditions.

### Molecular dynamics simulation (MD)

**Figure 11:**
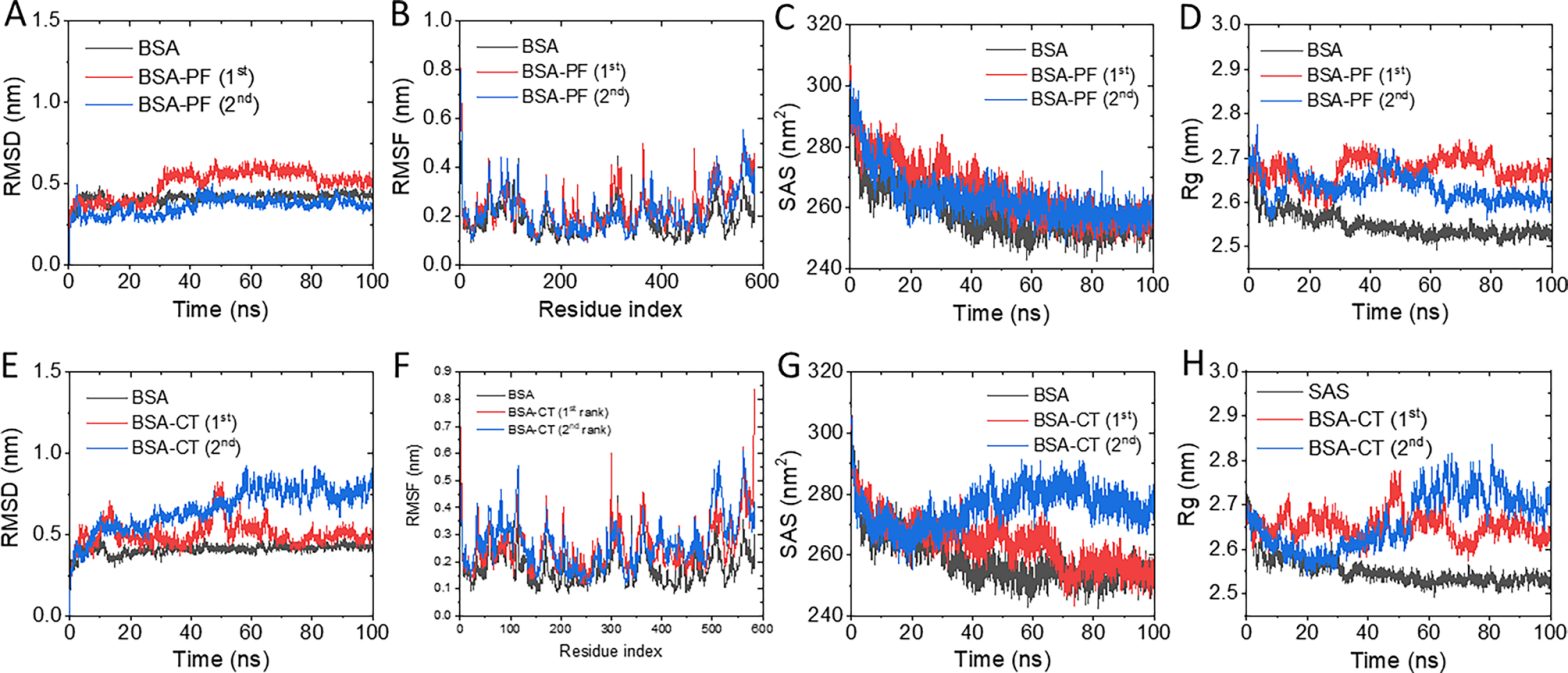
Molecular dynamic simulations of BSA‒PF (**A**-**D**) and BSA‒CT (**E**–**H**) complexes. Time course of the root mean square deviation (RMSD) of the C-alpha chain (**A**,**E**), root mean square fluctuation (RMSF) of the amino residue (**B**,**F**), solvent accessible surface (SAS; **C**,**G**), and radius of gyration (Rg; **D**,**H**) during the simulation time course were represented.

MD simulations were carried out for 100 ns time span to investigate the movement and stability of BSA‒pesticide complexes in comparison with the unbound BSA. The relative deviations of BSA’s C-alpha backbone from its initial conformation were observed in terms of root mean square deviation (RMSD). Unbound BSA gained a stable conformation with ~ 0.4 nm deviation after simulation for 5 ns (Fig. 11). However, upon complexation with the 1st and 2nd rank poses of PF and CT, the pesticide‒bound BSA took longer periods to attain stability, requiring at least 40 ns and 60 ns for BSA–PF (Fig. 11A) and BSA–CT (Fig. 11E) complexes, respectively. A plausible explanation was that the bindings of PF and CT may perturb the initial conformation of the BSA, which requires time to rearrange and stabilize its complex formation. In addition, the residue fluctuation patterns of BSA‒pesticide complexes were similar to those of the unbound BSA (Fig. 11B,F) even though some structural regions of the complexes exhibited a higher degree of deviation than that of the unbound BSA. This was especially true for subdomains IIIA and IIIB at residue positions ~ 380 to ~ 583, and the loop region of subdomain IIA at residue positions ~ 160 to ~ 190, indicating that these regions exhibit high flexibility and may not be directly involved in the binding process. A significant decrease in residue fluctuation was not observed in BSA‒pesticide complexes. However, the 1st rank poses of BSA‒PF and BSA‒CT exhibited identical root mean square fluctuation (RMSF) profiles with the unbound BSA at residue positions ~ 230 to ~ 265, belonging to the site I pocket, indicating the stable binding of PF and CT at this region. Likewise, the 2nd rank BSA‒PF and BSA‒CT showed similar RMSF with the unbound BSA at residue positions ~ 320 to ~ 370, suggesting the stable binding of PF and CT at the PC_lower_ region. The solvent accessible surfaces (SAS) of the unbound and pesticide-bound BSAs were decreased according to the simulation time and became steady at ~ 70 ns, except for the 2nd rank BSA–CT complex, as its SAS began to rise after decreasing for approximately 20 ns from the initial simulation (Fig. 11C,G). The bindings of PF and CT slightly produced higher SAS property of BSA as compared to the unbound BSA, where it was drastically observed on the 2nd rank BSA–CT complex. This finding is consistent with the estimated radius of gyration (Rg) parameters of the BSA-pesticide complexes that remained consistently higher than unbound BSA throughout the entire simulation (Fig. 11D,H), implying the decrease in protein structure compactness and stability upon complexation with pesticides.

## Conclusion

Mechanistic interactions between BSA and either PF or CT were obviously elucidated in this study by integrating various analytical approaches, such as fluorescence quenching, UV–Vis and CD spectroscopies, molecular docking analysis, and molecular dynamics simulation. The ADMET property predictions revealed that PF was inactive in many toxicity assessments, but its LD_50_ level was relatively low (162 mg/kg) as correlated with its experimentally and clinically proven neurotoxicity. However, CT is classified as a carcinogenic, mutagenic, and allergenic agent, but it exhibited low acute toxicity with a high LD_50_ value (7000 mg/kg). Fluorescence quenching and UV–Vis absorption analyses confirmed the phenomenon of interaction between BSA and PF or CT, which was mainly driven by the complex formation process. Thermodynamic model and molecular docking studies inferred that the BSA‒PF complex was geared by hydrophobic interaction, which was consensus with the previous reports on the interaction of serum albumin and other organophosphorus pesticides (e.g., chlorpyrifos, diazinon, parathion, and paraoxon)^45,46^. On the other hand, electrostatic interactions were the major binding modes of the BSA‒CT complex. The detailed mechanism of binding was elucidated by site probe competition, molecular docking, and molecular dynamics approaches. Both PF and CT were preferentially bound at the site I pocket of BSA. PF utilized its alkyl triphosphate groups to form hydrophobic bonds with Leu, Ile, Ala, and Arg residues near the site I of BSA, while CT exerted its tetrahydrophthalimide to bind with Tyr, Arg, and Ala residues. Therefore, PF and CT may compete with other therapeutic drugs and essential elements to sit in the binding pocket of serum protein, and in turn interfere with the pharmacokinetics and potency of other therapeutic drugs.

## Materials and methods

### Chemicals and reagents

PF and CT were obtained from Dr. Ehrenstorfer (Augsburg, Germany). BSA (98% purity without essential fatty acid), warfarin, and ibuprofen were purchased from Sigma-Aldrich (St. Louis, MO, USA). HPLC grade acetonitrile was obtained from Merck (Darmstadt, Germany) for preparation of PF and CT solutions. All other chemicals were analytical grade. BSA stock solution was prepared in Tris–HCl buffer (10 mM Tris–HCl and 150 mM NaCl, pH 7.4) and quantified by spectrophotometry at 280 nm with a molar extinction coefficient of 43,824 M^−1^ cm^−1^. All experiments were carried out with 2 μM BSA in Tris–HCl buffer containing 0.1% v/v acetonitrile, which was a carrier solvent for PF and CT.

### Steady-state fluorescence spectroscopy

The effects of pesticides on BSA fluorescence were monitored by a temperature-controlled QuantaMaster™ 40 spectrofluorometer (Photon Technology International, Inc., NJ, USA). The experiments were performed in triplicate. The spectroscopic parameters were experimentally stated as mean ± SD error. Briefly, different concentrations (0, 1, 5, 10, 20, 30, 40, 50, 60, and 80 µM) of PF or CT were independently incubated with BSA (2 µM) for 2 h at 290, 300, or 310 K, and then the fluorescence emission was recorded in a range between 285 and 450 nm wavelengths with a 280 nm excitation light. Likewise, site marker displacement analyses were carried out with the same procedure, while 2 µM of either warfarin or ibuprofen was co-incubated with the pesticide and BSA to serve as a molecular probe of the site I or site II drug-binding pocket, respectively. The inner filter effect was eradicated from the fluorescence data using Eq. (8).

8 $$F_{cor} = F_{obs} \times e^{{\frac{{(A^{ex} + A^{em} )}}{2}}}$$

$F_{cor}$ and $F_{obs}$ denote the corrected and measured fluorescence intensities of the sample, respectively. $A^{ex}$ and $A^{em}$ are the absorbances at excitation and emission wavelengths, respectively.

### UV–Vis absorption spectroscopy

Absorption spectra of BSA in the presence and absence of pesticide were measured by a NanoDrop One spectrophotometer (Thermo Fisher Scientific, MA, USA) with a quartz cuvette (1 cm path length). BSA concentration was kept constant at 2 µM, while varying PF or CT concentrations were at 0, 1, 20, 40, 60, and 80 µM.

### CD spectroscopy

CD spectra of BSA and BSA–pesticide complexes were measured by a Jasco J-815 spectropolarimeter (JASCO International Co. Ltd., Tokyo, Japan) using a 1 mm path length quartz cell flushed with nitrogen at 310 K. The BSA concentration remained constant at 2 μM, while PF or CT was varied to 5 and 50 μM. CD spectra were recorded in the range of 200–600 nm with a 60 nm/min scanning rate. CD data were analyzed with the BeStSel web server available at https://bestsel.elte.hu.

### In silico toxicokinetic prediction

Analogous to pharmacokinetics, the toxicokinetic properties of PF and CT were determined using web-based services, namely SwissADME (http://www.swissadme.ch)^47^, pkCSM (https://biosig.lab.uq.edu.au/pkcsm)^48^, and ProTox-II (https://tox-new.charite.de/protox_II)^49^. The chemical structures of PF and CT in SMILES format were used as inputs to execute the predictions by default settings.

### Molecular docking and molecular dynamics simulation

The BSA crystal structure (2.47 Å) was retrieved from the Protein Databank (PDB; https://www.rcsb.org) with an accession number of 4F5S, and it was cleaned to remove chain B, water molecules, and heteroatoms prior to performing molecular docking analyses. PF and CT structures were downloaded from the PubChem database (https://pubchem.ncbi.nlm.nih.gov) and later docked on the BSA structure using AutoDock Vina^50^ as an extension tool of UCSF Chimera 1.1.6 (https://www.cgl.ucsf.edu/chimera). The obtained binding poses of PF and CT on the BSA’s coordination were analyzed and visualized with UCSF Chimera and BIOVIA Discovery Studio visualizer (https://www.3ds.com).

The docking poses with the most favorable binding energy of PF and CT on BSA were selected for molecular dynamics simulation studies by WebGro^51^, a web-based GROMAC simulation tool available at https://simlab.uams.edu. The simulations were carried out for 100 ns using the GROMOS96 43a1forcefield with the SPC water model in a triclinic box solvated with 0.15 M NaCl at 310 K.

### Supplementary Information

Supplementary Information.

## Data Availability

All data analyzed during the current study are included in this published article and its Supplementary Information file; further inquiries can be directed to the corresponding author.
