# Supplementary material for: Revealing the mechanistic interactions of profenofos and captan pesticides with serum protein via biophysical and computational investigations
Source: Sci Rep. 2024 Jan 20;14:1788. doi: 10.1038/s41598-024-52169-2 (PMC10799918; doi:10.1038/s41598-024-52169-2)
Supplement: Supplementary file 1 — Supplementary Information. [file 41598_2024_52169_MOESM1_ESM.pdf]

Revealing the Mechanistic Interactions of Profenofos and Captan Pesticides with Serum Protein via Biophysical and Computational Investigations

Kamonrat Phopin[1,2], Waralee Ruankham[1], Supaluk Prachayasittikul[1], Virapong Prachayasittikul[2], Tanawut Tantimongcolwat[1,*]

[1]Center for Research Innovation and Biomedical Informatics, Faculty of Medical Technology, Mahidol University, Bangkok 10700, Thailand

[2]Department of Clinical Microbiology and Applied Technology, Faculty of Medical Technology, Mahidol University, Bangkok 10700, Thailand

Table S1. Estimated structural content (%) from CD spectra of BSA in the absence and presence of either PF or CT (20, 40, and 80 μM)

	**PF (μM)**				**CT (μM)**			
**% Content**	**0**	**20**	**40**	**80**	**0**	**20**	**40**	**80**
**Helix**	44.7	43.5	44.4	44	44.7	43.2	44.1	43
**Antiparallel**	1.8	3.2	1.8	1.6	1.8	1.6	2	3.9
**Parallel**	5.2	4.7	4.9	5.5	5.2	5.3	4.8	4.6
**Turns**	11.3	11.4	11.1	11.1	11.3	11.2	11.5	11.4
**Others**	37	37.2	37.8	37.9	37	38.7	37.6	37.1
**RMSD**	0.0039	0.0046	0.0042	0.0044	0.0043	0.0045	0.0048	0.0053

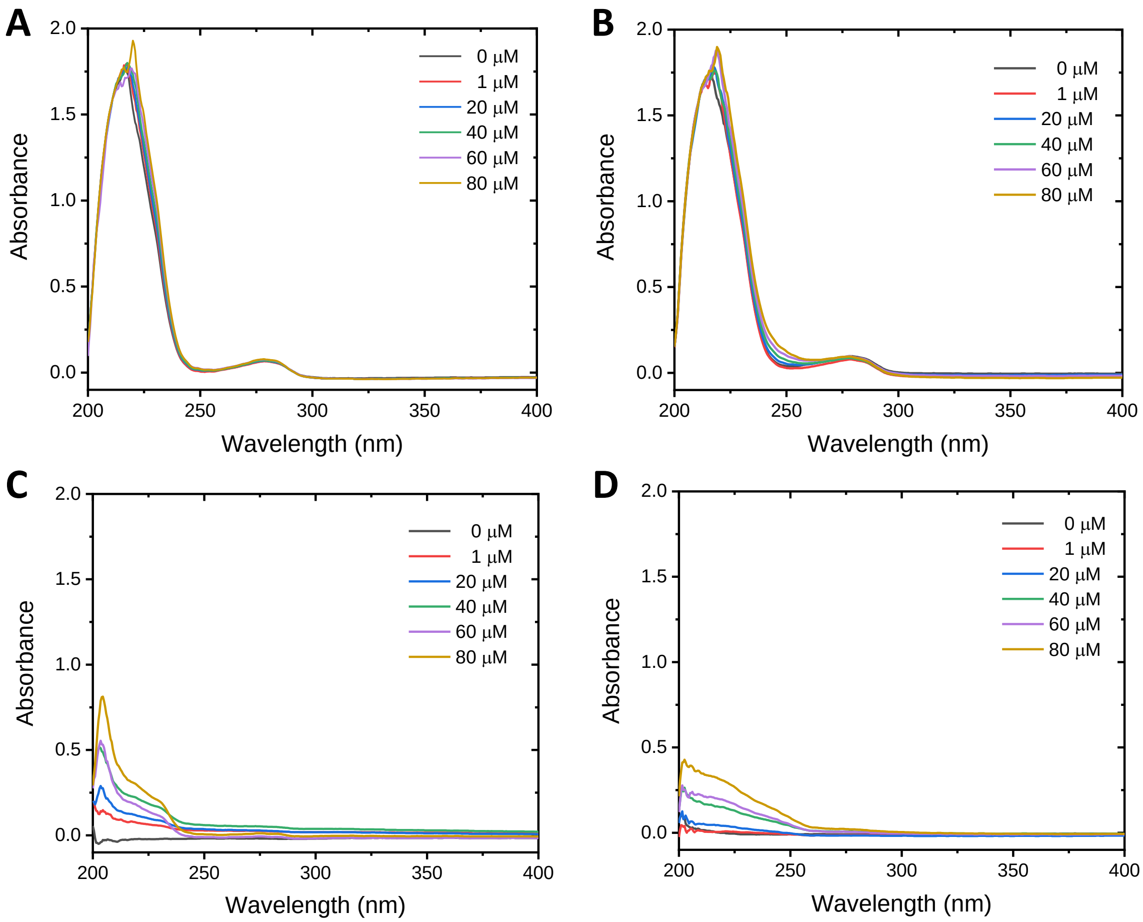

Figure S1. Absorption spectra at different concentrations of PF (**A** and **C**) or CT (**B** and **D**) in the absence (**C**, **D**) and presence of 2 μM BSA (**A**, **B**) in Tris buffer.

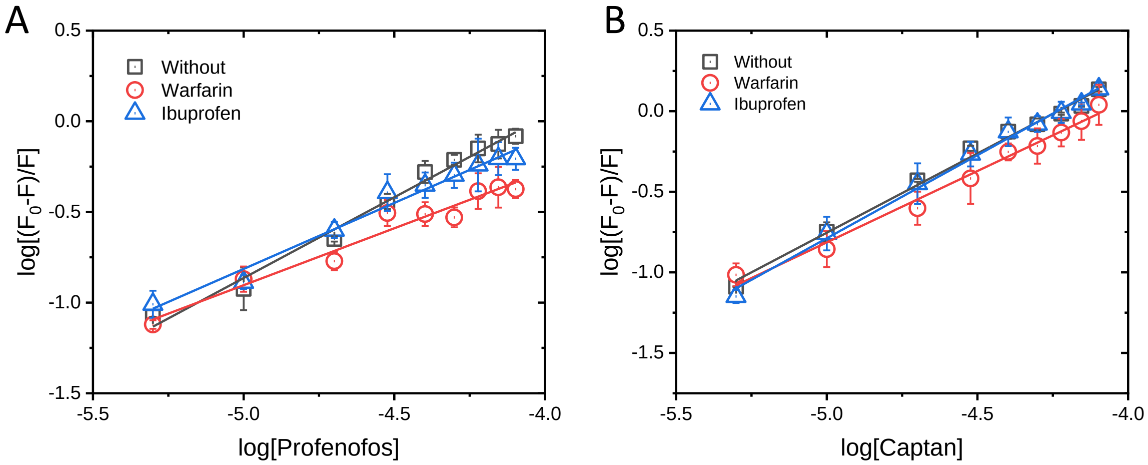

Figure S2. Double-logarithmic plots of BSA–PF (**A**) and BSA–CT (**B**) in the absence and presence of site probes at 310 K. Concentrations of BSA and site probe were equal at 2 μM, while PF or CT concentration was 5 μM.
